# Tele-consent using mixed reality glasses (NREAL) in pediatric inguinal herniorrhaphy: a preliminary study

**DOI:** 10.1038/s41598-022-06653-2

**Published:** 2022-02-24

**Authors:** Won-Gun Yun, Joong Kee Youn, Dayoung Ko, Inhwa Yeom, Hyun-Jin Joo, Hyoun-Joong Kong, Hyun-Young Kim

**Affiliations:** 1 Department of Pediatric Surgery, Seoul National University Hospital, Seoul, Republic of Korea; 2 Transdisciplinary Department of Medicine and Advanced Technology, Seoul National University Hospital, 101 Daehak-ro, Jongro-gu, Seoul, 03080 Republic of Korea; 3 Interdisciplinary Program in Bioengineering, Graduate School, Seoul National University, Seoul, Republic of Korea; 4 Institute of Medical and Biological Engineering, Medical Research Center, Seoul National University College of Medicine, Seoul, Republic of Korea; 5 Medical Big Data Research Center, Seoul National University College of Medicine, Seoul, Republic of Korea; 6 Department of Biomedical Engineering, Seoul National University College of Medicine, Seoul, Republic of Korea; 7 Department of Pediatric Surgery, Seoul National University College of Medicine, 101 Daehak-ro, Jongro-gu, Seoul, 03080 Republic of Korea

**Keywords:** Medical research, Biomedical engineering

## Abstract

There is an increasing demand and need for patients and caregivers to actively participate in the treatment process. However, when there are unexpected findings during pediatrics surgery, access restrictions in the operating room may lead to a lack of understanding of the medical condition, as the caregivers are forced to indirectly hear about it. To overcome this, we designed a tele-consent system that operates through a specially constructed mixed reality (MR) environment during surgery. We enrolled 11 patients with unilateral inguinal hernia and their caregivers among the patients undergoing laparoscopic inguinal herniorrhaphy between January through February 2021. The caregivers were informed of the intraoperative findings in real-time through MR glasses outside the operating room. After surgery, we conducted questionnaire surveys to evaluate the satisfaction and usefulness of tele-consent. We identified contralateral patent processus vaginalis in seven out of 11 patients, and then additionally performed surgery on the contralateral side with tele-consent from their caregivers. Most caregivers and surgeons answered positively about the satisfaction and usefulness of tele-consent. This study found that tele-consent with caregivers using MR glasses not only increased the satisfaction of caregivers and surgeons, but also helped to accommodate real-time findings by adapting surgical plan through the tele-consent.

## Introduction

When pediatric patients undergo surgery, some intraoperative findings may require giving explanations to and obtaining consent from caregivers. This situation occurs more often in pediatric surgeries than in adult surgeries; this is likely because pediatric surgery encompasses a large variety of disease groups, with most of them being benign diseases, so it is important to preserve the organs as much as possible during surgery^1^. Pediatric surgery can also be difficult because of limitations in pre-operative imaging due to radiation exposure, the need for sedation treatment, and the small body cavity.

Inguinal hernia is frequently encountered in children and requires surgery once diagnosed. Inguinal hernia can be diagnosed by confirming groin protrusion under high abdominal pressure, and the etiology of pediatric inguinal hernia is almost always patent processus vaginalis (PPV)^2^. An issue for several decades in pediatric inguinal hernia has been whether PPV was encountered incidentally during laparoscopic surgery should be repaired. Asymptomatic PPV develops into subsequent inguinal hernia later in only 10%; however, laparoscopic inguinal herniorrhaphy is associated with major complications such as nerve damage or infertility^3^. In the absence of unequivocal medical evidence, the opinion of caregivers may be more important.

In addition, in modern society, there is an increasing demand for patients and caregivers to actively participate in the treatment process and exercise their right to knowledge. Many studies have suggested that greater patient participation in medical decisions improves patients’ satisfaction^4^ and objective health outcomes^5^. The most difficult but essential factor in the process of involving patients and caregivers in the treatment process is obtaining informed consent. According to Cocanour et al.^6^, the three fundamental criteria for informed consent are that the patient or caregivers must be competent, adequately informed, and not coerced. In terms of adequately informed patients and caregivers, many studies have demonstrated that informed consent, which can involve improved readability through picture materials, etc., facilitates the understanding of patients and caregivers, their participation in the treatment process or clinical research, and the protection of autonomy^7–10^. As such, it is important to provide medical information in an easy-to-understand manner in the process of obtaining informed consent, but it is not easy to explain intraoperative findings to caregivers because there is a gap in medical information between surgeons and the caregivers, and the caregivers’ access to the operating rooms is restricted for reasons of contamination and security. Therefore, we propose a tele-consent method that can overcome spatial constraints and deliver intuitively unexpected intraoperative findings using mixed reality (MR) technology during surgery.

Recently, MR has emerged as a new technology that can be used in the field of surgery for training surgeons and patient education as well as in actual surgery. According to Alaker et al.^11^, practicing laparoscopic surgery through MR is not only effective, but is also associated with reduced error rates and improved tissue handling. There have been reports that applying MR in actual surgery leads to improvements in both treatment quality and patient outcomes^12,13^. However, to the best of the authors’ knowledge, no studies have reported tele-consent using MR. Therefore, tele-consent using MR technology represents a novel approach to obtain informed consent in tele-medicine^14^.

In this study, we developed an independent tele-consent platform involving MR glasses, real-time live-stream video, and a laparoscopic device that allows for the laparoscopic surgery scene to be shared with remote caregivers in real time, thus allowing them to have the intraoperative findings explained to them. We evaluated the usefulness of the tele-consent using mixed reality technology for caregivers and surgeons during pediatric inguinal herniorrhaphy.

## Results

### Demographics of patients and caregivers

The median age of patients at surgery was 3.7 years old and the male-to-female ratio was 2.7:1. Of the 11 patients, PPV of the contralateral side was confirmed in seven patients during the operation, and the caregivers of these patients all desired and agreed to proceed with the surgery on contralateral side. The mean operation time was 34.5 ± 8.50 min (mean ± standard deviation) and no immediate adverse events occurred in any patient (Table 1).

**Table 1:** Demographics of patients.

Variables	N = 11
**Age at surgery (year)**	
Median (range)	3.7 (0.1–8.5)
**Male to female ratio**	2.7
**Height at surgery (cm)**	
Median (range)	99.4 (50.6–130.6)
**Body weight at surgery (kg)**	
Median (range)	15.4 (4.3–30.6)
**Location of inguinal hernia at initial presentation**	
Rt	5
Lt	6
**No. of contralateral PPVs found during surgery**	
Rt. inguinal hernia → Lt. PPV	4
Lt. inguinal hernia → Rt. PPV	3
**Operation time (min)**	
Mean (SD)	34.5 (8.50)
**Immediate adverse event**	0

*Lt.* left, *Rt.* Right, *PPV* patent processus vaginalis.

The median age of the caregivers was 39 (37.5–41.5) years old [median (interquartile range)]; all caregivers were the patient’s parents. In terms of socioeconomic status, 10 caregivers (90.9%) were college graduates and nine caregivers (81.8%) had middle-income status (Table 2).

**Table 2:** Demographics of caregivers who responded to the survey.

Variable	N = 11
**Age (year)**	
Median (IQR)	39 (37.5–41.5)
**Male to female ratio**	0.38
**Relationship**	
Father (%)	3 (27.3)
Mother (%)	8 (72.7)
**Education level**	
High school (%)	1 (9.1)
College (%)	9 (81.8)
Graduation (%)	1 (9.1)
**Economic status**	
Middle income (%)	9 (81.8)
Refused to respond (%)	2 (18.2)

*IQR* interquartile range.

### Survey results after the tele-consent process

The results of the caregivers’ survey are shown in Tables 3 and 4. Caregivers were not familiar with MR (1.8 ± 0.75); however, they responded positively regarding the process of communication with medical staff using MR glasses (4.5 ± 0.24, 89.1% ± 4.81).

**Table 3:** Results based on survey of caregivers’ tele-consent.

Variable	Mean	SD	Min	Max
**Experience with MR**				
I am familiar with MR	1.8	0.75	1	3
**Communication with medical staff using MR glasses**				
Communication with the medical staff was comfortable	4.6	0.50	4	5
I received information from the medical staff	4.2	0.40	4	5
The medical staff was confident in the treatment plan	4.5	0.52	4	5
Total average	4.5	0.24	4.2	4.6
**Satisfaction with MR glasses**				
Next time in the same situation, I would like to hear the non-face-to-face explanation as it was in this case, rather than the old way	4.1	0.54	3	5
Overall, I was satisfied with the new device	4.6	0.50	4	5
I will recommend the new device to my family or friends	4.4	0.50	4	5
Total average	4.4	0.27	4.1	4.6
**Effect of MR glasses on treatment**				
Time was saved by using the device	4.5	0.52	4	5
The disease was properly treated	4.7	0.47	4	5
Anxiety about limited access to the medical environment was reduced	4.5	0.52	4	5
Total average	4.6	0.10	4.5	4.7
**Ease of using MR glasses**				
It was not difficult to use the device	4.6	0.50	4	5
There was no reluctance to use the device	4.7	0.47	4	5
The display worked properly	4.5	0.52	4	5
The data processing speed was fast	4.8	0.40	4	5
The device was ready quickly	4.2	0.60	3	5
The device worked fine overall	4.8	0.40	4	5
Total average	4.6	0.24	4.2	4.8
**Overall satisfaction**	4.7	0.47	4	5
**Total usefulness**	4.5	0.12	4.4	4.6

*MR* mixed reality, *SD* standard deviation, *Min* minimum, *Max* maximum.

**Table 4:** Caregivers’ usefulness ratings of the tele-consent using mixed reality glasses, based on communication, satisfaction, effect on treatment, ease of using.

Variable	Mean% (± SD)	Median% (min–max)
Communication	89.1 (4.81)	90.9 (83.6–82.7)
Satisfaction	87.9 (4.58)	87.3 (83.6–92.7)
Effect on treatment	90.3 (2.10)	90.9 (90.3–94.5)
Ease of using	92.4 (4.80)	93.6 (83.6–96.4)

Subscale scores are presented as percentages of the maximum possible score (5 points for 11 caregivers, 55 points).

*SD* standard deviation, *Min* minimum, *Max* maximum.

Most caregivers responded that they agreed or strongly agreed with the questions involving satisfaction with using MR glasses (4.4 ± 0.27, 87.9% ± 4.58) and expressed overall satisfaction (4.7 ± 0.47). Of the 11 caregivers, 10 agreed or strongly agreed to ‘Next time in the same situation, I would like to hear the non-face-to-face explanation as it was in this case, rather than the old way’. Also, caregivers responded positively about the effect of MR glasses (4.6 ± 0.10, 90.3% ± 2.10) on treatment and ease of using MR glasses (4.6 ± 0.24, 92.4% ± 4.80). All caregivers scored 4 or more out of 5 in response to the item, ‘Anxiety about limited access to the medical environment was reduced.

The survey results involving surgeons are presented in Tables 5 and 6. Surgeons were familiar with MR (4.3 ± 0.58). Further, surgeons answered positively to most of the questions about communication with caregivers using MR glasses (4.8 ± 0.38, 95.6% ± 7.70), satisfaction with MR glasses (4.8 ± 0.50, 95.0% ± 10.00), the effect of MR glasses on treatment (4.7 ± 0.58, 93.3% ± 11.55), and the ease of using MR glasses (4.4 ± 1.11, 88.9% ± 22.11). The tele-consent platform had no network failure or system error during the study, however, one in three surgeons reported that tele-consent device required a lot of learning about setting up and using.

**Table 5:** Results of surgeon survey following tele-consent.

Variable	Mean	SD	Min	Max
**Experience with MR**				
I am familiar with MR	4.3	0.58	4	5
**Communication with caregivers using MR glasses**				
Communication with caregiver was comfortable	4.3	0.58	4	5
I was able to provide sufficient information to the caregiver	5.0	0.00	5	5
The non-face-to-face communication method using the new device was satisfactory	5.0	0.00	5	5
Total average	4.8	0.38	4.3	5.0
**Satisfaction with MR glasses**				
The device system was suitable for medical communication	4.0	1.73	2	5
Overall, I was satisfied with the non-face-to-face explanation process	5.0	0.00	5	5
I would like to continue the non-face-to-face explanation method in the future	5.0	0.00	5	5
I think this device will be helpful for medical communication in the future	5.0	0.00	5	5
Total average	4.8	0.50	4.0	5.0
**Effect of MR glasses on treatment**				
Time was saved by using the device	4.0	1.73	2	5
The non-face-to-face explanation helped to form a rapport with the caregiver	5.0	0.00	5	5
The disease was properly treated	5.0	0.00	5	5
Total average	4.7	0.58	4.0	5.0
**Ease of using MR glasses**				
It was not difficult to use the device	5.0	0.00	5	5
There was no reluctance to use the device	5.0	0.00	5	5
A lot of learning was required to use the device	2.7	0.58	2	3
The display worked properly	5.0	0.00	5	5
The microphone and speaker worked properly	5.0	0.00	5	5
The data processing speed was fast	5.0	0.00	5	5
The device preparation process was not difficult	2.3	1.15	1	3
Overall, the device was convenient to use	5.0	0.00	5	5
There was no increase in physical fatigue after non-face-to-face communication	5.0	0.00	5	5
**Total average**	4.4	1.11	2.3	5.0
**Total usefulness**	4.7	0.15	4.4	4.8

*MR* mixed reality, *SD* standard deviation, *Min* minimum, *Max* maximum.

**Table 6:** Surgeons’ usefulness ratings of the tele-consent using mixed reality glasses, regarding communication, satisfaction, effect on treatment, ease of using.

Variable	Mean% (± SD)	Median% (min–max)
Communication	95.6 (7.70)	100.0 (86.7–100.0)
Satisfaction	95.0 (10.00)	100.0 (80.0–100.0)
Effect on treatment	93.3 (11.55)	100.0 (80.0–100.0)
Ease of using	88.9 (22.11)	100.0 (46.67–100.0)

Subscale scores are presented as percentages of the maximum possible score (5 points for 3 surgeons, 15 points).

*SD* standard deviation, *Min* minimum, *Max* maximum.

## Discussion

This study was performed to investigate the usefulness of tele-consent for both caregivers and surgeons using MR glasses in pediatric laparoscopic herniorrhaphy. Survey results regarding the usefulness of the tele-consent system using MR glasses showed a positive opinion of both caregivers and surgeons (total usefulness: 4.5 ± 0.12, caregivers; 4.7 ± 0.15, surgeons). Both caregivers and surgeons responded positively in all four sub-categories (communication, satisfaction, effect on treatment, ease of use) related to usefulness. These results clearly suggest that the tele-consent system using MR glasses is a promising strategy in actual clinical practice.

In modern society, there is a social atmosphere wherein patients and caregivers actively participate in this treatment process. Considering this trend, doctors are increasingly communicating with patients and caregivers to include them in the treatment process. However, when doctors talk with patients and caregivers about their medical conditions, it is difficult for them to convey complex medical information because of the disparity between their medical knowledge and that of the general public. Of course, in recent years, with the continued development of various technologies, the general public can now easily access medical information through videos and medical theses if they choose^15^. Nevertheless, it is still difficult for patients and caregivers to fully understand intraoperative findings due to limited access and medical knowledge.

Recently, many efforts have been made to apply MR technology to the medical field. Augmented reality (AR) combines three-dimensional (3D) computer-generated objects and text superimposed onto real images and videos, all in real-time. The main difference between virtual reality (VR) and AR is that the latter uses real images, video frames, and 3D graphics alone^16^. It is becoming increasingly difficult to train young surgical residents or students in real clinical settings due to the increased emphasis on safety-related problems in patients and rapid advances in various surgical techniques such as minimal invasive surgery^17^. Therefore, many companies and hospitals are conducting research on MR medical device development with a focus on the educational aspect^18^. It is also gradually being used in actual surgery^19^. In addition, MR technology has the possibility to be used in other aspects like tele-medicine than those mentioned.

Therefore, we applied a specially constructed MR environment including MR glasses, real-time live-stream video, and a laparoscopic device to enable real-time communication and tele-consent with caregivers in response to the need for changes in the surgical plan during surgery. This special MR environment was completely different from AR or VR, as remote caregivers could share in the laparoscopic scene with the surgeon and hear the explanation in real time simply by wearing MR glasses. This method has many advantages over the traditional face-to-face explanation in various aspects. First, caregivers have substantially increased access to information related to the operating room than they previously did. From a caregiver’s perspective, the conventional method simply consists of indirect hearing about intraoperative findings from the surgeon, and this method may also cause contamination and delays in surgery that can be harmful to the patient. By contrast, the method of remote explanations using MR glasses not only avoid the aforementioned problems, but also has advantages in terms of enabling sufficient understanding and immersion for caregivers. Many studies have reported that using 3D visualization instead of 2D leads to increased immersion. According to Kim et al.^20^, the immersion offered by 3D optics may help improve performance in the operation room, thus allowing surgeons to be more focused on the care and have greater depth acuity. Further, from the surgeon’s point of view, it is an opportunity to explain any surgical findings to caregivers with less time and effort and to enhance the legitimacy of surgeon’s medical decision.

Tele-consent has been successfully implemented in several clinical trials. In previous studies, tele-consent was used to obtain consent for clinical research using a software solution without MR^21^. Our study differed from previous studies in that we used a specially constructed MR environment to facilitate the caregiver’s understanding of surgery, and that this system was used in actual clinical situations. Real-time communication practices via MR technology in the medical field, such as tele-consent decisions successfully utilized in this study and further large-scale follow-up studies can lead to clinical applications in other clinical settings. In particular, it is expected that this technology will become more popular when it is difficult to visit hospitals or other countries under restricted conditions such as during the era of the severe acute respiratory syndrome coronavirus 2 (SARS-CoV-2) pandemic^22^. For example, emergency procedures are often required in intensive care units, where there are many urgent situations. In these cases, tele-consent through MR technology may be a solution to the difficulty involved in obtaining the caregiver’s consent before conducting an invasive procedure. From the caregiver’s perspective, this method can also help them understand the patient’s rapidly changing medical condition and the need for an invasive procedure. Daniel et al.^23^ suggested that there are many possible uses of tele-medicine in intensive care units beyond the examples mentioned earlier. In addition, according to Doruk et al.^24^, the burden of disease and disparities in access to surgical care for children in low and middle-income countries is of great importance to the global pediatric surgery community, and this issue deserves greater attention. It is expected that the introduction of MR technology will provide sufficient medical information to people in low and middle-income countries by overcoming distance barriers. In addition to the operating room, patients and caregivers in intensive care units or regions that medical services is underserved may be able to receive assistance through this system. We are also considering follow-up studies related to various other diseases and inter-professional discussions. Due to legal regulations, it is currently difficult to use this method in practice. Thus, appropriate legal issues need to be considered in further technological developments.

In this study, we successfully investigated the usefulness of tele-consent with 11 caregiver-participant pairs and three surgeons. However, our study had limitations. First, this study was performed in single center, and the number of subjects in this study was small. Second, this study did not establish a control group, and only investigated patients exposed to MR, and therefore the study was not compared with the conventional method. Third, a new technology always appears promising, but its cost-effectiveness needs to be thoroughly verified before its widespread implementation. The current version of the system, being a first-generation prototype, requires technical assistants in setting up the instrumentation, mainly to secure surgeons from possible contamination. To minimize the needs of technical intervention, we envisage a future study to enhance cost-effectiveness and contamination management, as well as the overall usability and user experience. The operating room is one of the most important and costly environments in health care, so decision-makers must balance the potential for improving care using the latest technology with any additional costs^25^. Fourth, this system has a long learning curve for installation and application, based on at least one physician response to the survey. In our study, medical assistants outside the surgical field assisted with the logistics of this technology. However, to prevent a crowded operating room, the development of a coordinated system with an intuitive user interface within the surgical field will be needed in the future.

## Conclusion

Real-time sharing of intraoperative findings with remote caregivers using a specially constructed MR environment not only increased the satisfaction of caregivers and surgeons, but also helped real-time findings be accommodated in adapting surgical plans through the tele-consent process.

## Methods

### Study design and subjects

In this study, we have performed cross-sectional survey of caregivers and surgeons to evaluate the usefulness of a tele-consent system using MR glasses for the caregivers who received the service and the surgeons who provided the service.

This study involved 11 patients and their caregivers in Seoul National University Children Hospital between January through February 2021. The study included patients who were younger than 18 years old, diagnosed with unilateral inguinal hernia, and consented to undergo laparoscopic inguinal herniorrhaphy, and patients and/or caregivers who agreed to participate in this study. Informed consent was obtained from all subjects and/or their legal caregivers. Patients who were older than 18 years old and had an emergency event during surgery were excluded.

The system developer and two technical assistants set up the tele-consent platform both in the surgery room and waiting room. In the surgery room, the WebRTC-based Cart was connected with video inputs, including a webcam for a laparoscopic video via HDMI-to-USB converter. Medical staff in the operating room could use a shortcut on the WebRTC Cart to connect with the platform. For MR glasses in the waiting room, a shortcut that redirects caregivers to the platform was pre-installed. The procedure in each room took less than 5 min in total.

In addition, three pediatric surgeons who had abundant experience of laparoscopic inguinal herniorrhaphy in this medical center participated in this study. When the surgery started with the insertion of trocars, the operator evaluated the presence or absence of patent processus vaginalis (PPV) on the side that was not diagnosed with an inguinal hernia. After confirming the presence or absence of PPV, operator in the operating room and caregivers in the waiting room where was only occupied by the caregivers and research personnel for privacy and confidentiality were connected via our designed communication platform and MR glasses, which showed the laparoscopic view in real-time. The operator’s verbal explanation on PPV and proceeding operations was transmitted from the embedded microphone of WebRTC-based Cart system to the speaker installed inside the temples of MR glasses, which the caregiver was wearing in the waiting room. The operator could also use visual annotation function of the tele-consent platform, such as marking the PPV location on the laparoscopic video feed by simply speaking and carrying on the surgery without having to touch any contaminated items such as cart systems outside the sterile surgical field. The caregivers and operator discussed any necessary changes to the surgical plan and agreed to another surgical procedure, thereby providing tele-consent. Throughout the study, two technical assistants facilitate technical operations, including resolving any network failure or issues associated with low battery power.

### Tele-consent platform (Fig. 1)

**Figure 1:**
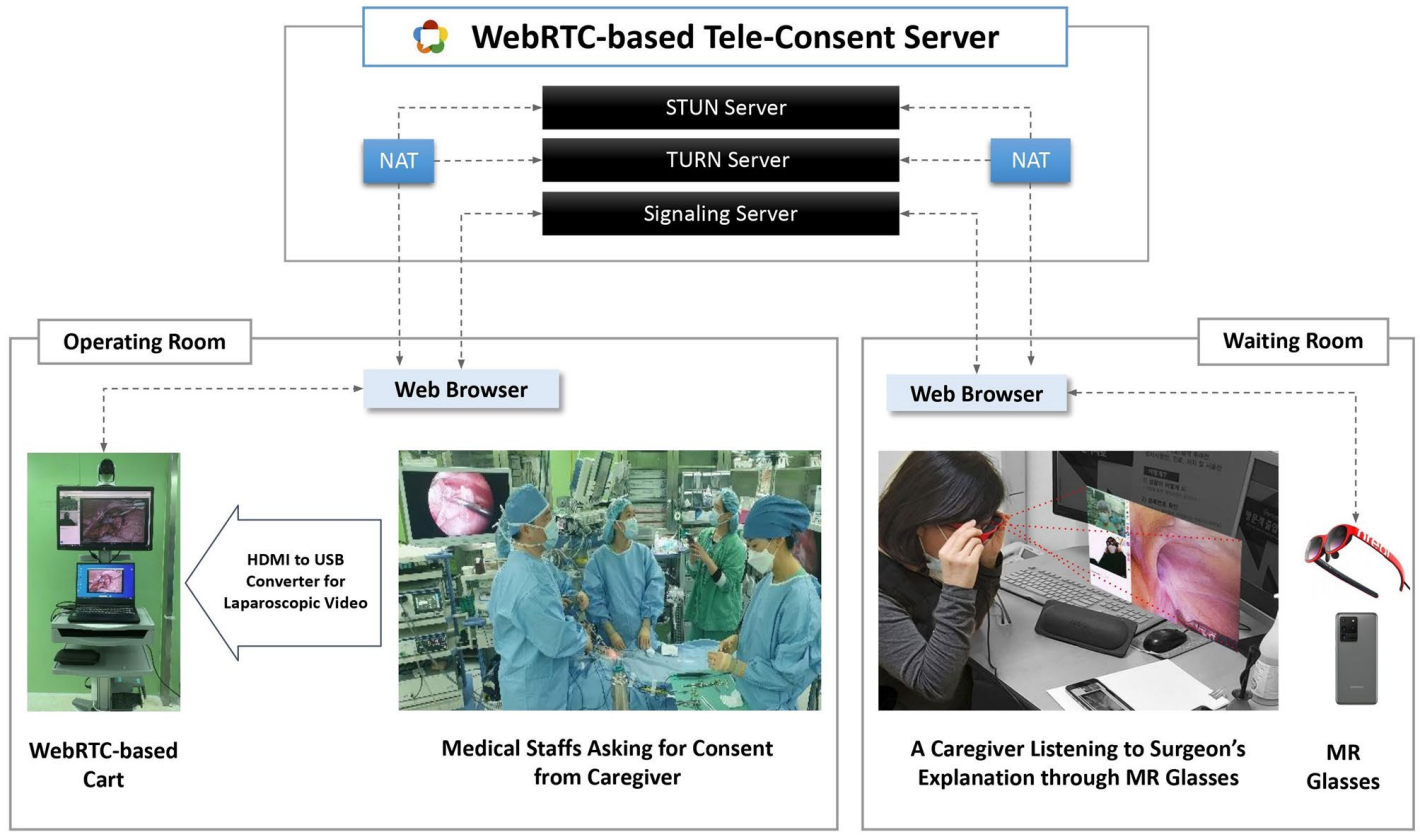
A tele-consent platform: A specially-constructed communication environment consisting of a laptop, a 360° camera, laparoscopy video, and mixed reality glasses connected by WebRTC servers.

To enhance the caregivers’ access to information, and to facilitate the consent procedures in the midst of an ongoing surgery, we propose a tele-consent platform. This platform was designed to connect caregivers in the waiting room and surgeons in the operating room, respectively with MR glasses and medical computer cart. The major functionalities of the platform, accessible from both the MR glasses and cart are as follows: (1) a video call between caregivers and surgeons, (2) live-streaming of the laparoscopy procedure, and (3) a drawing feature for annotating on the live-streamed video. Once the platform is accessed on the web via a medical computer cart, surgeons can instantiate a virtual scene where the aforementioned screens and functionalities appear. The caregivers can then join the scene via their MR glasses, with the scene being augmented on the glasses. In this setting, the caregivers can access the real-time visual and voice information on the ongoing surgical process; verbally and/or gesturally express their consent while communicating with surgeons through the video call feature. The surgeons, on the other hand, can provide audio-visual explanations on the status quo of surgery, using the live streaming of laparoscopy and the annotation feature as visual aids.

For the system deployment, we leveraged NREAL Light for wearable the MR glasses, and a custom-built operating server system and website using Web Real-Time Communication (WebRTC), an open-source technology that works across various operating systems (e.g., Windows, Android, and iOS) and web browsers (e.g., Chrome and Firefox)^25^. The system was developed using Jquery 3.3.1 based on NodeJS 8.11.3 and RTCMultiConnection 3.5.1 libraries on a Microsoft Windows Server 2016 standard operating system. To perform WebRTC real-time communication, it is necessary to exchange information and find users through Signaling, Session Traversal Utilities for NAT (STUN), and Traversal Using Relays around NAT (TURN). In this study, information was exchanged on the media format and negotiations were made in the process of exchanging messages between devices through a signaling server using socket.IO. WebRTC uses state-of-the-art encryption standards (Hypertext Transfer Protocol Secure, Datagram Transport Layer Security, and Secure Real) to provide health insurance portability and accountability compliance security for data transmission.

The WebRTC Cart System sent HDMI signals to the laptop via a video capture card, which was output through a light source device (CV-170) for OLYMPUS endoscopy. The software was accessed via a web browser (Google Chrome v89) on the Microsoft Windows Server 10 operating system. A PTZ camera (RS-1260HD) was installed at the top of the WebRTC Cart system, thus allowing the medical staff to share a video of the desired operating field situation through Zoom (maximum 12 × magnification) while using the angle deformation (maximum 355°) function of the camera. MR scenes were displayed to the users through the connection of NREAL glasses and a Smartphone (LG V50S ThinQ), and they were accessed via a web browser (Google Chrome v89) with Android 10. Real-time sharing of the intraoperative findings took place in an environment where a wireless network access (100 Mbps) was provided.

### Outcomes

We collected data on demographics of all patients and caregivers who participated in this study, including operation time and immediate adverse events such as bleeding, nerve injury, infertility and atelectasis or apnea related with general anesthesia. We administered questionnaires to capture the caregivers’ opinions between the completion of surgery and patient discharge from the hospital. Surgeons filled out a questionnaire after completing all the surgeries of enrolled patients. The caregivers’ questionnaire consisted of 17 multiple-choice five point Likert-scale questions and the surgeons’ questionnaire consisted of 20 multiple-choice five point Likert-scale questions, with the following main topics: (1) previous experience and familiarity with MR, (2) communication with medical staff or caregivers using MR glasses, (3) satisfaction with MR glasses, (4) effect of MR glasses on treatment, (5) ease of using MR glasses, and (6) overall satisfaction. Five response options were provided for each question as follows: 1 = strongly disagree, 2 = disagree, 3 = neither, 4 = agree, and 5 = strongly agree. The caregivers’ questionnaire was created by modifying part of the questionnaire using a paper survey of patients undergoing dermatology tele-consultation using MedX^26^. The questionnaire for surgeons was created with reference to existing studies discussing evaluated new technologies^27–29^.

### Statistical analyses

All statistical analyses were conducted using IBM SPSS statistics software for Windows, version 25.0 program (IMB Corp., Armonk, NY, USA). Variables related to patient and caregiver demographics were expressed as median with range. Surgery time was expressed as mean with standard deviation. We expressed survey results as mean score with standard deviation, and additionally analyzed the scores for each subcategory of survey; communication, satisfaction, effect on treatment, ease of use. This study conformed to the ethical guidelines of the 1975 Helsinki Declaration and was approved by the Institutional Review Board of Seoul National University Hospital (IRB No. 2101-084-118).
